# Neural model for learning-to-learn of novel task sets in the motor domain

**DOI:** 10.3389/fpsyg.2013.00771

**Published:** 2013-10-22

**Authors:** Alexandre Pitti, Raphaël Braud, Sylvain Mahé, Mathias Quoy, Philippe Gaussier

**Affiliations:** ETIS Laboratory, UMR CNRS 8051, the University of Cergy-Pontoise, ENSEACergy-Pontoise, France

**Keywords:** task sets, fronto-parietal system, decision making, incremental learning, cortical plasticity, error-reward processing, gain-field mechanism, tool-use

## Abstract

During development, infants learn to differentiate their motor behaviors relative to various contexts by exploring and identifying the correct structures of causes and effects that they can perform; these structures of actions are called *task sets* or *internal models*. The ability to detect the structure of new actions, to learn them and to select on the fly the proper one given the current task set is one great leap in infants cognition. This behavior is an important component of the child's ability of learning-to-learn, a mechanism akin to the one of intrinsic motivation that is argued to drive cognitive development. Accordingly, we propose to model a dual system based on (1) the learning of new task sets and on (2) their evaluation relative to their uncertainty and prediction error. The architecture is designed as a two-level-based neural system for context-dependent behavior (the first system) and task exploration and exploitation (the second system). In our model, the task sets are learned separately by reinforcement learning in the first network after their evaluation and selection in the second one. We perform two different experimental setups to show the sensorimotor mapping and switching between tasks, a first one in a neural simulation for modeling cognitive tasks and a second one with an arm-robot for motor task learning and switching. We show that the interplay of several intrinsic mechanisms drive the rapid formation of the neural populations with respect to novel task sets.

## 1. Introduction

The design of a multi-tasks robot that can cope with novelty and evolve in an open-ended manner is still an open challenge for robotics. It is however an important goal (1) for conceiving personal assistive robots that are adaptive (e.g., to infants, the elderly and to the handicapped people) and (2) for studying from an inter-disciplinary viewpoint the intrinsic mechanisms underlying decision making, goal-setting and the ability to respond on the fly and adaptively to novel problems.

For instance, robots cannot yet reach the level of infants for exploring alternative ways to surmount an obstacle, searching for a hidden toy in a new environment, finding themselves the proper way to use a tool, or solving a jigsaw puzzle. All these tasks require to be solved within boundaries of their given problem space, without exploring it entirely. Thus, robots lack this ability to detect and explore new behaviors and action sequences oriented toward a goal; i.e., what is called a *task set* (Harlow, 1949; Collins and Koechlin, 2012).

The ability to manipulate dynamically task sets is however a fundamental aspect of cognitive development (Johnson, 2012). Early in infancy, infants are capable to perform flexible decision-making and dynamic executive control even at a simple level in order to deal with the unexpected (Tenenbaum et al., 2011). Later on, when they are more mature, they learn to explore the tasks space, to select goals and to focus progressively on tasks of increasing complexity. One example in motor development is the learning of different postural configurations. Karen Adolph explains for instance how infants progressively differentiate their motor behaviors into task sets (i.e., the motor repertoire) and explore thoroughly the boundaries of each postural behavior till becoming expert on what they discover (Adolph and Joh, 2005, 2009). Adolph further argues that the building of a motor repertoire is not preprogrammed with a specific developmental timeline but that each postural behavior can be learned independently as separated tasks without pre-ordered dependencies to the other ones (crawling, sitting, or standing).

This viewpoint is also shared by neurobiologists who conceive the motor system to structure the actions repertoire into “internal models” for each goal to achieve (Wolpert and Flanagan, 2010; Wolpert et al., 2011). Each novel contextual cue (e.g., handling a novel object) promotes the acquisition and the use of a distinct internal model that does not modify the existing neural representations used to control the limb on its own (White and Diedrichsen, 2013). Moreover, each task set is evaluated depending on the current dynamics and on the current goal we want to perform (Orban and Wolpert, 2011). For instance, we switch dynamically from different motor strategies to the most appropriate one depending on the context; e.g., tilting the racket to the correct angle in order to give the desired effect on the ball, or for executing the proper handling of objets with respect to their estimated masses (Cothros et al., 2006).

From a developmental viewpoint, the capability for flexible decision-making gradually improves in 18 months-old infants (Tenenbaum et al., 2011). Decision-making endows infants to evaluate the different alternatives they have for achieving one goal with respect to the ongoing sequence and to select the correct one(s) among different alternatives. It owes them also the possibility to inhibit some previously learned strategies in order to explore new ones never seen before (Yokoyama et al., 2005).

IN AI, this craving to explore, to test and to embed new behaviors is known as intrinsic motivation (Kaplan and Oudeyer, 2007). In Kaplan and Oudeyer's words: “The idea is that a robot (…) would be able to autonomously explore its environment not to fulfill predefined tasks but driven by some form of intrinsic motivation that pushes it to search for situations where learning happens efficiently”. In this paper, we focus more on the idea that the rewards are self-generated by the machine itself (Singh et al., 2010) and that the function of intrinsic motivation is mainly to regulate the exploration/exploitation problem, driving exploratory behavior and looking for different successful behaviors in pursuing a goal. In that context, we propose that the ability to choose whether or not to follow the same plan or to create a novel one out of nothing—in regard to the current situation—is an intrinsic motivation. We studied for instance the role of the neuromodulator acetylcholine in the hippocampus for novelty detection and memory formation (Pitti and Kuniyoshi, 2011).

Meanwhile, the capability to make decision and to select between many options is one important aspect of intrinsic motivation because otherwise the system would be only passive and would not be able to select or encourage one particular behavior. Taking decisions in deadlock situations requires therefore some problem-solving capabilities like means-end reasoning (Koechlin et al., 2003) and error-based learning capabilities (Adolph and Joh, 2009). For instance, means-end reasoning and error-based learning are involved in some major psychological tests such as the Piagetian “A-not-B error test” (Diamond, 1985; Smith et al., 1999; Schöner and Dineva, 2007), Harlow's learning set test (Harlow, 1949) and tool-use (Lockman, 2000; Fagard et al., 2012; Vaesen, 2012; Guerin et al., 2013). The A-not-B error test describes a decision-making problem where a 9-month old infant still pertains to select an automatic wrong response (e.g., the location *A*) and cannot switch dynamically from this erronous situation to the correct one (e.g., the location *B*). Above this age, however, infants do not make the error and switch rapidly to the right location. A similar observation is found in Harlow's experiments on higher learning (Harlow, 1949) where Rhesus monkeys and humans have to catch the pattern of the experiment in a series of learning experiences. Persons and monkeys demonstrate that they learn to respond faster when facing a novel and similar situation by switching to the correct strategy, by catching the pattern to stop making the error: they show therefore that they do not master isolated tasks but, instead, they grasp the relation between the events. In one situation, if the animal guessed wrong on the first trial, then it should switch directly to the other solution. In another situation, if it guessed right on the first trial, then it should continue. This performance seems to require that the monkey, the baby or the person use an abstract rule and solve the problem with an apparent inductive reasoning (Tenenbaum et al., 2011). In line with these observations on the development of flexible behaviors, researchers focused on tool-use: when infants start to use an object as a means to an end, they serialize their actions toward a specific goal, as for example reaching a toy with a stick (Fagard et al., 2012; Rat-Fischer et al., 2012; Guerin et al., 2013). Tool-use requires also finding patterns like the shape of grasping, order and sequentiality of patterns (Cothros et al., 2006).

Considering the mechanisms it may involve, Karen Adolph emphasizes the ability of *learning-to-learn* (Adolph and Joh, 2005), a process akin to Harlow (1949). Harlow coined the expression to distinguish the means for finding solutions to novel problems from simple associative learning and stimulus generalization (Adolph, 2008). Adolph reinterprets this proposal and suggests that two different kinds of thinking and learning are at work in the infant brain, governing the aspects of exploration and of generalization (Adolph and Joh, 2009). On the one hand, one learning system is devoted to the learning of task sets from simple stimulus-response associations. For instance, when an infant recognizes the context, he selects his most familiar strategy and reinforces it within his delimiting parameter ranges. On the other hand, a second learning is devoted to detect a new situation as is and to find a solution dynamically in a series of steps. Here, the acceptance of uncertainty gradually leads for making choices and decisions in situation never seen before. However, which brain regions and which neural mechanisms this framework underlies?

Among the different brain regions, we emphasize that the post-parietal cortex (PPC) and the pre-frontal cortex (PFC) are found important (1) for learning context-dependent behavior and (2) for evaluating and selecting these behaviors relative to their uncertainty and error prediction. Regarding the PPC, different sensorimotor maps co-exist to represent structured information like spatial information or the reaching of a target, built on coordinate transform mechanisms (Stricanne et al., 1996; Andersen, 1997; Pouget and Snyder, 2000). Furthermore, recent studies acknowledge the existence of context-specific neurons in the parieto-motor system for different grasp movements (Brozovic et al., 2007; Andersen and Cui, 2009; Baumann et al., 2009; Fluet et al., 2010). Regarding the PFC, Johnson identifies the early development of the pre-frontal cortex as an important component for enabling executive functions (Johnson, 2012) while other studies have demonstrated difficulty in learning set formation following extensive damage of the prefrontal cortex (Warren and Harlow, 1952; Yokoyama et al., 2005). The PFC manipulates information on the basis of the current plan (Fuster, 2001), and it is active when new rules need to be learned and other ones rejected. Besides, its behavior is strongly modulated by the anterior cingulate cortex (ACC) which plays an active role for evaluating task sets and for detecting errors during the current episode (Botvinick et al., 2001; Holroyd and Coles, 2002; Khamassi et al., 2011). If we look now at the functional organization of these brain structures, many authors emphasize the interplay between an associative memory of action selection in the temporal and parietal cortices (i.e., an integrative model) and a working memory for actions prediction and decision making in the frontal area (i.e., a serial model) (Fuster, 2001; Andersen and Cui, 2009; Holtmaat and Svoboda, 2009). All-in-all, these considerations permit us to draw a scenario based on a two complementary learning systems.

**Figure 1:**
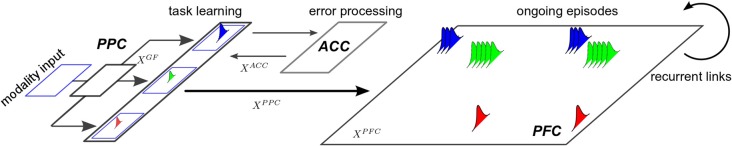
**Framework for task set selection**. The whole system is composed of three distinct neural networks, inspired from Khamassi et al. (2011). The PPC network conforms to an associative network. It binds the afferent sensory inputs from each other and map them to different motor outputs with respect to a task set. The ACC system is a error-based working memory that processes the incoming PPC signals and feeds back an error to them with respect to current task. This modulated signal is used to tune the population of neurons in PPC by reinforcement learning, it is also conveyed to the PFC map, which is a recurrent network that learns dynamically the spatio-temporal patterns of the ongoing episodes with respect to the task.

More precisely, we propose to model a dual system based on (1) the learning of task sets and on (2) the evaluation of these task sets relative to their uncertainty, and error prediction. Accordingly, we design a two-level based neural system for context-dependent behavior (PPC) and task exploration and prediction (ACC and PFC); see Figure 1. In our model, the task sets are learned separately by reinforcement learning in the post parietal cortex after their evaluation and selection in the prefrontal cortex and anterior cyngulate cortex. On the one hand, the learner or agent stores and exploits its familiar knowledge through a reinforcement learning algorithm into contextual patterns called and collected from all its different modalities. On the other hand, the learner evaluates and compares the way it learns, and selects the useful strategies while it discards others or tests new ones on the fly if no relevant strategy is found. We perform two different experimental setups to show the sensorimotor mapping and switching between tasks, one in a neural simulation for modeling cognitive tasks and another with an arm-robot for motor task learning and switching. We use neural networks to learn simple sensorimotor mapping for different tasks and compute their variance and error for estimating the sensorimotor prediction. Above a certain threshold, the error signal is used to select and to valuate the current strategy. If no strategy is found pertinent for the current situation, this corresponds to a novel motor schema that is learned independently by a different map. In a cognitive experiment similar to Harlow (1949) and Diamond (1990), we employ this neural structure to learn multiple spatio-temporal sequences and switch between different strategies if an error has occurred or if a reward has been received (error-learning). In a psycho-physic experiment similar to Wolpert and Flanagan (2010), we show how a robotic arm learns the visuomotor strategies for stabilizing the end-point of its own arm when it moves it alone and when it is holding a long stick. Here, the uncertainty on the spatial location of the end-point triggers the decision-making from the two strategies by selecting the best one given the proprioceptive and visual feedback and the error signal delivered.

## 2. Materials and methods

In this section, we present the neural architecture and the mechanisms that govern the dynamics of the neurons, of reinforcement learning and of decision-making. We describe first the bio-inspired mechanism of rank-order coding from which we derive the activity of the parietal and of the pre-frontal neurons. In second, we describe the reinforcement learning algorithm, the error prediction reward and the decision-making rules.

### 2.1. PPC—gain-field modulation and sensorimotor mapping

**Figure 2:**
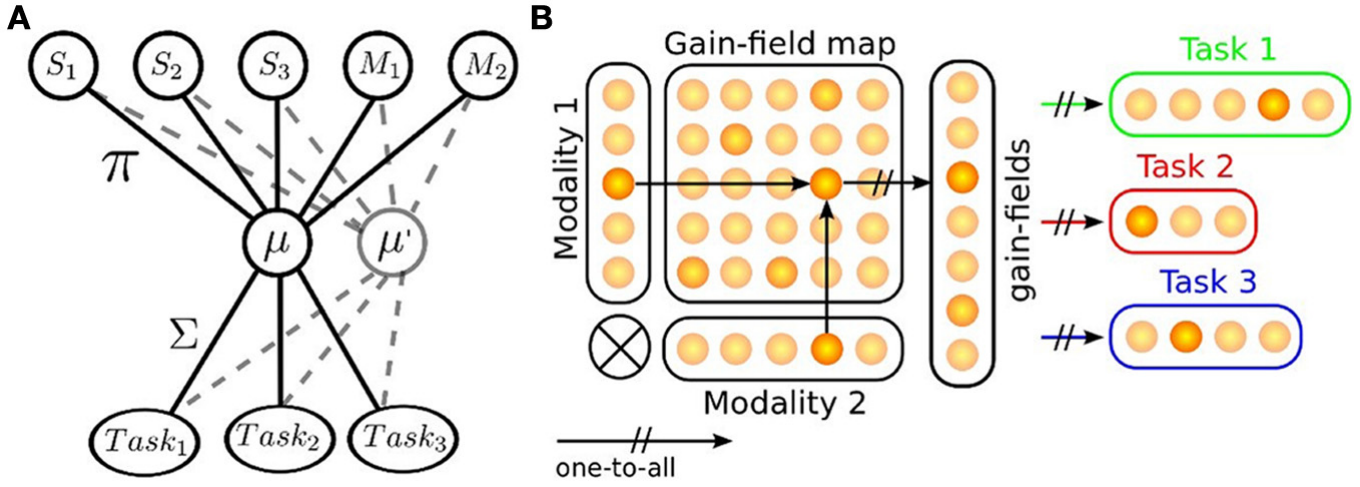
**Task sets mapping, the mechanism of gain-fields. (A)** Gain-fields neurons are units used for sensorimotor transformation. They transform the input activity into another base, which is then fed forward to various outputs with respect to their task. Gain-fields can be seen as meta-parameters that decrease the complexity of the sensory-motor problem into a linear one. **(B)** example of GF neurons sensorimotor transformation for two modalities projecting to three different task sets; each GF neuron contributes to one particular feature of the tasks (Pouget and Snyder, 2000; Orban and Wolpert, 2011).

We employ the rank-order coding neurons to model the sensorimotor mapping between input and output signals with an architecture that we have used in a previous research (Pitti et al., 2012). This architecture implements multiplicative neurons, called gain-field neurons, that multiply unit by unit the value of two or more incoming neural populations, see Figure 2. Its organization is interesting because it transforms the incoming signals into a basis functions' representation that could be used to simultaneously represent stimuli in various reference frames (Salinas and Thier, 2000). The multiplication between afferent sensory signals in this case from two population codes, *X*_*m*_1__ and *X*_*m*_2__, {*m*_1_, *m*_2_ ∈ *M*_1_, *M*_2_}, produces the signal activity *X*_*n*_ to the *n* gain-field neurons, *n* ∈ *N*:

(1) $$X^{GF}=X^{M_{1}}\times X^{M_{2}}$$

The key idea here is that the gain-field neurons encode two information at once and that the amplitude of the gain-field neurons relates the values of one modality *conditionally* to the other; see Figure 2A. The task is therefore encoded into a space of lower dimension (Braun et al., 2009, 2010). We exploit this feature to model the parietal circuits for different contextual cues and internal models, which means that, after the encoding, the output layers learn the receptive fields of the gain-field map and translates this information into various gain levels. In Figure 2B, we give a concrete example of one implementation, here delineated to two modalities, with *N* gain-fields projecting to three different tasks set of different size. We explain thereinafter (1) how the gain fields neurons learn the associations between various modalities and (2) how the neurons of the output map learn from the gain fields neurons for each desired task.

### 2.2. Rank-order coding algorithm

**Figure 3:**
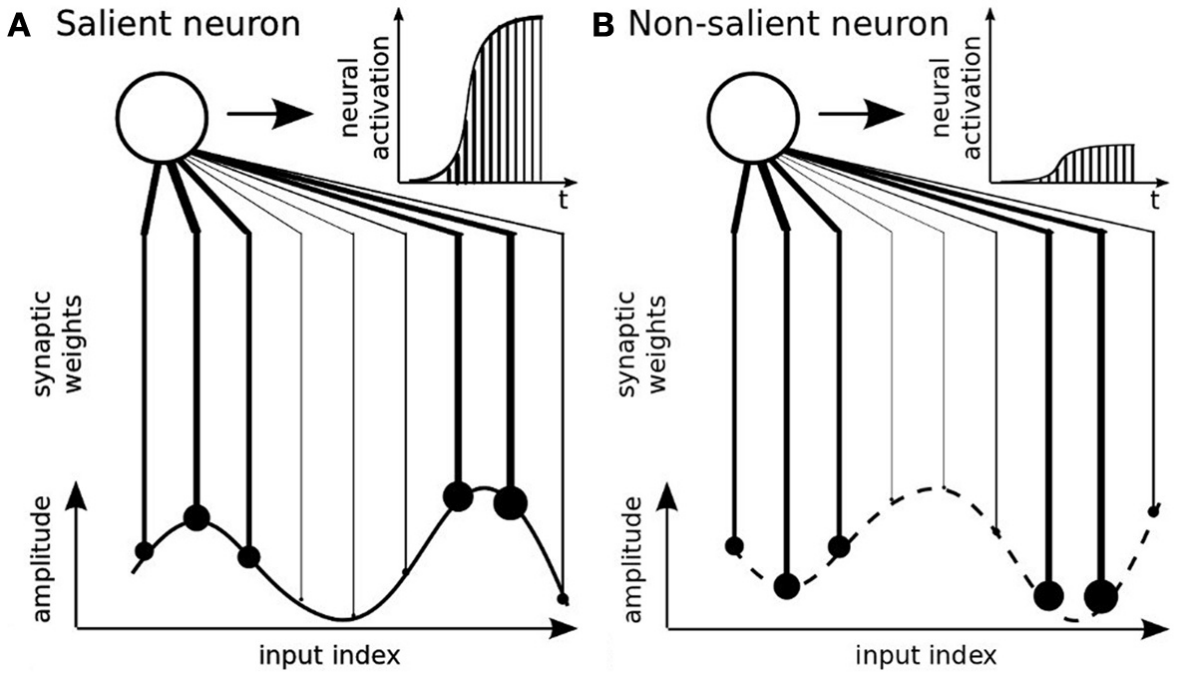
**Rank-Order Coding principle (Thorpe et al., 2001)**. This type of neuron encodes the rank code of an input signal. Its amplitude is translated into an ordered sequence and the neuron's synaptic weights are associated to this sequence. The neural activity is salient to this particular order only, see **(A)**, and otherwise not, see **(B)**.

We implement a hebbian-like learning algorithm proposed by Van Rullen et al. (1998) called the Rank-Order Coding (ROC) algorithm. The ROC algorithm has been proposed as a discrete and faster model of the derivative integrate-and-fire neuron (Van Rullen and Thorpe, 2002). ROC neurons are sensitive to the sequential order of the incoming signals; that is, its *rank code*, see Figure 3A. The distance similarity to this code is transformed into an amplitude value. A scalar product between the input's rank code with the synaptic weights furnishes then a distance measure and the activity level of the neuron. More precisely, the ordinal rank code can be obtained by sorting the signals' vector relative to their amplitude levels or to their temporal order in a sequence. We use this property respectively for modeling the signal's amplitude for the parietal neurons and the spatio-temporal patterns for the prefrontal neurons. If the rank code of the input signal matches perfectly the one of the synaptic weights, then the neuron fully integrates this activity over time and fires, see Figure 3A. At contrary, if the rank order of the signal vector does not match properly the ordinal sequence of the synaptic weights, then integration is weak and the neuron discharges proportionally to it, see Figure 3B.

The neurons' output *X* is computed by multiplying the rank order of the sensory signal vector *I*, *rank*(*I*), by the synaptic weights *w*; *w* ∈ [0, 1]. For a vector signal of dimension *M* and for a population of *N* neurons (*M* afferent synapses), we have for the GF neurons and for the output PPC neurons:

(2) $$\{\begin{array}{ll} X_{n}^{GF} & =\sum_{m\;\in \;M}\frac{1}{rank(I_{m})}w_{n,\;m}^{GF-Modality}\\ X_{n}^{PPC} & =\sum_{m\;\in \;M}\frac{1}{rank(I_{m})}w_{n,\;m}^{PPC-GF} \end{array}$$

The updating rule of the neurons' weights is similar to the winner-takes-all learning algorithm of Kohonen's self-organizing maps (Kohonen, 1982). For the best neuron *s* ∈ *N* and for all afferent signals *m* ∈ *M*, we have for the neurons of the output layer:

(3) $$\{\begin{array}{ll} w_{s,\;m}^{PPC-GF} & =w_{s,\;m}^{PPC-GF}+\Delta w_{s,\;m}^{PPC-GF}\\ \Delta w_{s,\;m}^{PPC-GF} & =\frac{1}{rank(I_{m})}-w_{s,\;m}^{PPC-GF}, \end{array}$$

the equations are the same for GF neurons (not reproduced here). We make the note that the synaptic weights follow a power-scale density distribution that makes the rank-order coding neurons similar to basis functions. This attribute permits to use them as receptive fields so that the more distant the input signal is to the receptive field, the lower is its activity level; e.g., Figure 3B.

### 2.3. Reinforcement learning and error reward processing

The use of the rank-order coding algorithm provides an easy framework for reinforcement learning and error-based learning (Barto, 1995). For instance, the adaptation of the weights in Equation 3 can be modified simply with a variable α ∈ [0, 1] that can ponder Δ*w*; see Equation 4. If α = 0, then the weights are not reinforced: *W*_*t* + 1_ = *W*_*t*_. If α = 1, then the weights are reinforced in the direction of Δ*W*: *W*_*t* + 1_ = *W*_*t*_ + αΔ*W*. In addition, conditional learning can be made simply by summing an external bias β to the neurons output *X*. By changing the amplitude of the neurons, we change also the rank-order to be learned and influence therefore the long-term the overall organization of the network; see Equation 5.

(4) Δw ←αΔw, α∈[0,1]

(5) X ←X+β, β∈[−1,+1]

#### 2.3.1. Cortical plasticity in PPC

For modeling the cortical plasticity in the PPC output maps, we implement an experience-driven plasticity mechanism. Observations done in rats show that during the learning of novel motor skills the synapses rapidly spread in the neocortex immediately as the animal learns a new task (Xu et al., 2009; Ziv and Ahissar, 2009). Rougier and Boniface proposed a dynamic learning rule in self-organizing maps to combine both the stability of the synapses' population to familiar inputs and the plasticity of the synapses' population to novel patterns (Rougier and Boniface, 2011). In order to model this feature in our PPC map, we redefine the coefficient α in Equation 5 and we rearrange the formula proposed by Rougier and Boniface:

(6) $$\alpha =e^{1/\eta^{2}/||max(X^{PPC})-X_{s}^{PPC}||}\in [0,1]$$

where η is the elasticity or plasticity parameter that we set to 1 and *max*(*X*^*PPC*^) is the upper bound of the neural activity, its maximal value, whereas *max*(*X*^*PPC*^) is the current maximum value within the neural population, with α = 0 when *X*^*PPC*^_*s*_ = *max*(*X*^*PPC*^). In this equation, the winner neuron learns the data according to its own distance to the data. If the winner neuron is close enough to it, it converges slowly to represent the data. At contrary, if the winner neuron is far from the data, it converges rapidly to it.

#### 2.3.2. Error-reward function in ACC

For modeling ACC, we implement an error-reward function similar to Khamassi et al. (2011) and to Q-learning based algorithms. The neurons' value is updated afterwards only when an error occurs, then a ihnibitory feedback error signal is sent to the winning neuron to diminish its activity *X*_*win*_: *ACC*(*X*_*win*_) = −1; the neurons equation *X* is updated as follows:

(7) $$X_{n}^{PPC}=\sum_{m\;\in \;M}\frac{1}{rank(I_{m})}w_{n,\;m}+ACC(X_{n}^{PPC}).$$

The neurons activity in *ACC* is cleared everytime the system responds correctly or provides a good answer. ACC can be seen then as a contextual working memory, a saliency buffer extracted from the current context when errors occur inhibiting the wrong actions performed. Its activity may permit to establish an exploration-based type of learning by trial and errors and an attentional switch signal from automatic responses, in order to deal with the unexpected when a novel situation occurs.

### 2.4. PFC—spatio-temporal learning in a recurrent network

We can employ the rank-order coding for modeling spike-based recurrent neural network in which the amplitude values of the incoming input signals are replaced by its past spatio-temporal activity pattern. Although the rank-order coding algorithm has been used at first to model the fast processing of the feed-forward neurons in *V*1, its action has been demonstrated to replicate also the hebbian learning mechanism of Spike Timing-Dependent Plasticity (STDP) in cortical neurons (Bi and Poo, 1998; Abbott and Nelson, 2000; Izhikevich et al., 2004). For a population of *N* neurons, we arbitrarily choose to connect each neuron to a buffer of size 20 × *N* so that they encode the rank code of the neurons amplitude value over the past 20 iterations. At each iteration, this buffer is shifted to accept the new values of the neurons.

(8) $$X_{n}^{PFC}=\sum_{m\;\in \;M}\frac{1}{rank(buffer_{m})}w_{n,\;m}+X_{n}^{PPC}.$$

Recurrent networks can generate novel patterns on the fly based on their previous activity pattern while, at each iteration, a winning neuron gets its links reinforced. Over time, the system regulates its own activity whereas coordinated dynamics can be observed. These behaviors can be used for anticipation and predictive control.

## 3. Results

We propose to study the overall behavior of each neural system during the learning of task sets and the dynamics of the ensemble working together. The first three experiments are performed in a computer simulation only. They describe the behavior of the PPC maps working solely, working along the ACC system and working along the ACC and PFC systems for learning and selecting context-dependent task sets. Experiment 4 is performed on a robot arm. This experiment describes the acquisition and the learning of two different task set during the manipulation or not of a tool.

### 3.1. Experiment 1—plasticity vs stability in learning task sets

**Figure 4:**
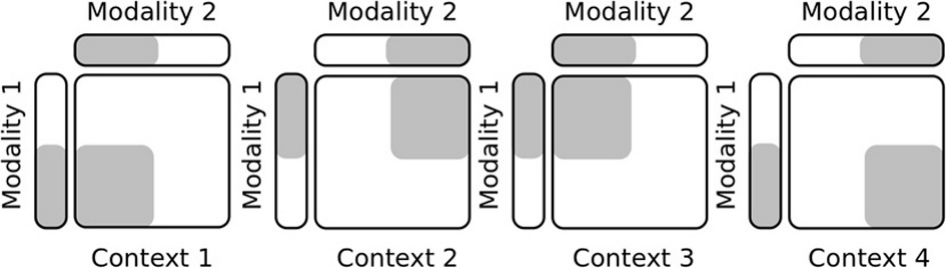
**Protocol setup in task sets learning**. This simple protocol explains how the experimental setup is done for acquiring different contexts incrementally and for selecting them.

In this first experiment, we test the capabilities of our network to learn incrementally novel contexts without forgetting the older ones, which corresponds to the so-called plasticity/stability dilemma of a memory system to retain the familiar inputs as well as to incorporate flexibly the novel ones. Our protocol follows the diagram in Figure 4 in which we show gradually four different contexts for two input modalities with vectors of ten indices. The input patterns are randomly selected from an area in the current context chosen randomly and for a period of time also variable. In this experiment, the PPC output map has 50 neurons that receive the activity of twenty gain-fields neurons, see Figure 2B.

**Figure 5:**
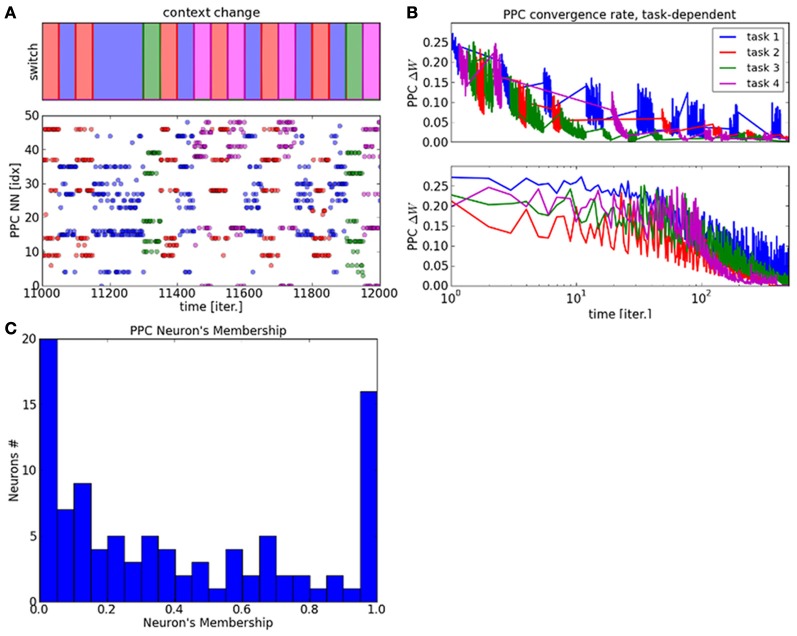
**Raster plot of the PPC output map and plasticity vs. stability within the map. (A)** the graph displays the neural dynamics during task switch among four different contexts. **(B)** Convergence rate of the PPC network with respect to each task. **(C)** The degree of plasticity and stability within the PPC output map is represented as the probability distribution of the neurons membership to the cluster relative to a context. This histogram shows two behaviors within the system. On the one hand, one third of the neurons present very stable dynamics with membership to one context only. On the other hand, two third of the neurons are part of different clusters and therefore to different contexts. The later neurons follow a power law distribution showing very plastic dynamics.

We display in Figure 5A the raster plot of the PPC neurons' dynamics with distinct colors with respect to the context. Contexts are given gradually, one at a time, so that some neurons have to unlearn their previous cluster first in order to fit the new context. It is important to note that categorization is unsupervised and decided due to the experience-driven plasticity rule in Equation 6. In order to demonstrate the plasticity of the PPC network during the presentation of a new context, we present the context number four, plotted in magenta and never seen before, at *t* = 11500. Here, the new cluster is rapidly formed and stable over time due again to the cortical plasticity mechanism from Equation 6. The graph displays therefore not only the plasticity of the clusters in the PPC network but also their robustness.

This property is also shown in Figure 5B where the convergence rates of the PPC weights vary differently for each task. This result explains how the PPC self-organizes itself into different clusters that specialize flexibly with respect to the task. The ratio between stability and plasticity in shown in Figure 5C within the network with the histogram of the neuron's membership over a certain time interval. The stability of one neuron is computed as its probability distribution relative to each context. The higher values correspond to very stable neurons, which are set to one context only and do not deviate from it, whereas the lower values correspond to very flexible neurons that change frequently context from one to another.

The histogram shows two probability distributions within the system and therefore two behaviors. For the neurons corresponding to values near the strong peak at 1.0, their activity is very stable and strongly identified to one context. This shows that for one third of the neurons, the behavior of the neural population is very stable. At reverse, the power law curve centered on 0.0 shows the high variability of certain neurons, which are very dynamic for one third of the neural population.

**Figure 6:**
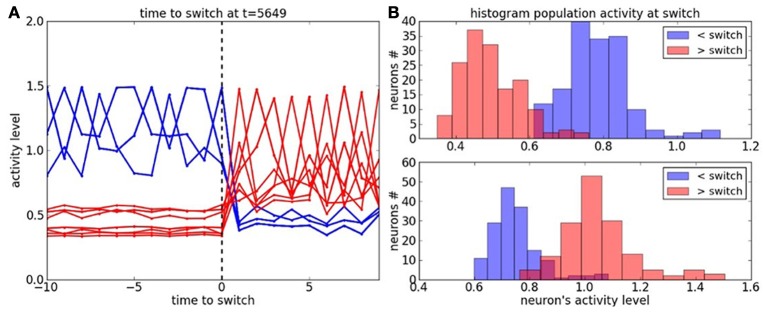
**Cluster dynamics at the time to switch. (A)** Neural dynamics of the active clusters before and after the switch; resp. in blue and in red. **(B)** Histogram of the neural population at the time to switch with respect to the active clusters before and after the switch.

We study now the neurons' activity during a task switch in Figure 6. In graph **(A)**, the blue lines correspond to the neurons' dynamic belonging to the context before the switch and the red lines correspond to the neurons' dynamic belonging to the context after the switch. The activity level in each cluster is very salient for each context. The probability distribution of the neurons' dynamic, with respect to each context is plotted in Figure 6B. It shows a small overlap between the contexts before and after the switch.

### 3.2. Experiment 2—learning task sets with a reinforcement signal

In this second experiment, we reproduce a decision-making problem similar to those done in monkeys and humans with multiple choices and rewards (Churchland and Ditterich, 2012). The rules are not given in advance and the tasks switch randomly after a certain period of time with no regular pattern. The goal of the experiment is to catch the input-output correspondence pattern to stop making the error. The patterns are learned dynamically by reinforcement learning within each map and should ideally be done without interference from each other. The error signal indicates when an input-output association is erronous with respect to a hidden policy, however, we make the note that it does not provide any hint about how to minimize the error. To understand how the whole system works, we focus our experiment on the PPC network with the ACC error processing system first, then with the PFC network. We choose to perform a two-choices experiment, with two output PPC maps initialized with random connections from the PPC map. The PPC network consists therefore of the gain-field architecture with the two output maps for modeling the two contexts. The two maps are then bidirectionally linked to the ACC system; the input signals for modality 1 and 2 are projected to the PPC input vectors of twenty units each; map1 has twelve output units and map2 has thirteen output units and project to ACC of dimension twenty-five units.

The hidden context we want the PPC maps to learn is to have output signals activated for specific interval range of the inputs signals, namely, the first output map has to be activated when input neurons of indices below ten are activated, and reciprocally, the second output map has to be activated when input neurons of indices above ten are activated—this corresponds to the two first contexts in Figure 4. The error prediction signal is updated anytime a mistake has been done on the interval range to learn. As expressed in the previous section, the ACC error signal resets always its activity when the PPC maps start to behave correctly.

**Figure 7:**
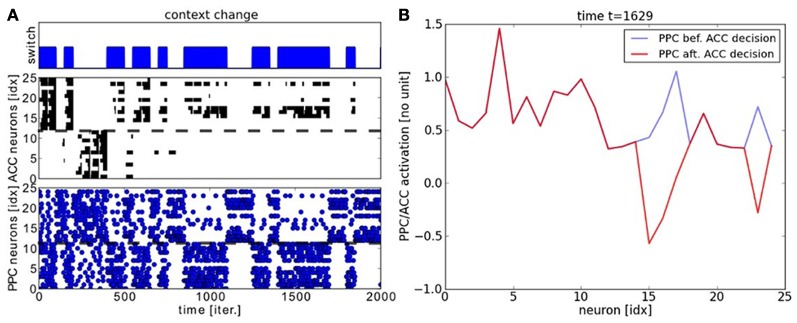
**Experiment on two-choices decision making and task switching. (A)** Neural dynamics of PPC neurons and ACC error system during task switch. We plot in the chart in the top the temporal interval for each task. Below the, neural dynamics of the PPC maps and in the middle, its erronous activity retranscribed in the ACC system. ACC works as a working memory that keep tracks of the erronous outputs, which is used during the learning stage. ACC is reset each time the PPC system gives a correct answer. Through reinforcement learning, the PPC maps converge gradually to the correct probability distribution. **(B)** Snapshot of the PPC maps in blue modulated negatively by ACC in red.

We analyze the performance of the PPC-ACC system in the following. We display in Figures 7A,B the raster plots of the PPC and ACC dynamics with respect to the context changes for different periods of time. The chart on the top displays the timing for context switch, the chart on the middle plots the ACC system working memory and the chart below plots the output of the PPC units. The Figure 7A is focusing on the beginning of the learning phase and the Figure 7B when the system has converged. We observe from these graphs that the units of the output maps self-organize very rapidly to avoid the error. ACC modulates negatively the PPC signals. We make the note that the error signal does not explicitly inhibit one map or the other but only the wrongly actived neuron of the map. As it can be observed, over time, each map specializes to its task. As a result, learning is not homogenous and depends also to the dimension of the context; that is, each map learns with a different convergence rate. ACC error rapidly reduces its overall activity for the learning of task1 with respect to map1, although the error persists for the learning of task2 with respect to map2 where some neurons still fires wrongly.

**Figure 8:**
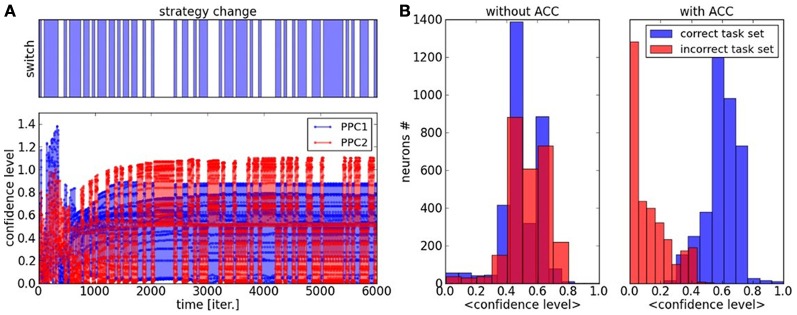
**Confidence Level of PPC maps during task switch, dynamics and histogram. (A)** The confidence level is the difference between the amplitude of most activated neuron and the second one within each map. After one thousand iterations, the two maps rapidly specialize their dynamics to its associated task. This behavior is due to the ACC error-based learning. **(B)** histogram of the probability distribution of the confidence level with and without ACC. With ACC, we observe a clear separation in two distributions, which correspond to a decrease of uncertainty with respect to the task. In comparison, the confidence level in an associative network without an error feedback gives a uniform distribution.

We propose to study the convergence of the two maps and the confidence level of the overall system for the two tasks. We define a confidence level index as the difference of amplitude between the most active neurons in map1 and map2. We plot its graph in Figure 8 where the blue color corresponds to the confidence level for task1 with *v*_*s_map1*_ − *v*_*s_map2*_ and the color red corresponds to the confidence level for task2 with *v*_*s_map2*_ − *v*_*s_map1*_ during the learning phase. The dynamics reproduce similar trends from Figure 7 where the confidence level constantly progresses till convergence to a stable performance rate, with a threshold around 0.4 above which a contextual state is recognized or not. Before 1000 iterations, the maps are very plastic so the confidence level fluctuates rapidly and continuously between different values but at the end of the learning phase, the maps are more static so the confidence level appears more discrete. This state is clearly observable from the histogram of the confidence level plotted on the right in Figure 8B for the case where the ACC error signal is injected to the associative network. The graph presents a probability distribution with two bell-shaped centred on 0.1 and 0.7, which corresponds to the cases of recognition or not of the task space. In comparison, the probability distribution for the associative learning without error-feedback is uniform, irrespective to the task; see Figure 8B in blue.

### 3.3. Experiment 3—adaptive learning on a temporal sequence based on error prediction reward

We attempt to replicate now Harlow's experiments on adaptive learning, but, in comparison to the previous experiments, it is the temporal sequence of task sets that is taken into account for the reward. We employ our neural system in a cognitive experiment first to learn multiple spatio-temporal sequences and then to predict when a change of strategy has occurred based on the error or on the reward received. With respect to the previous section also, we add the PFC-like recurrent neural network to learn the temporal sequence from the PPC and ACC signals, see Figure 1.

**Figure 9:**
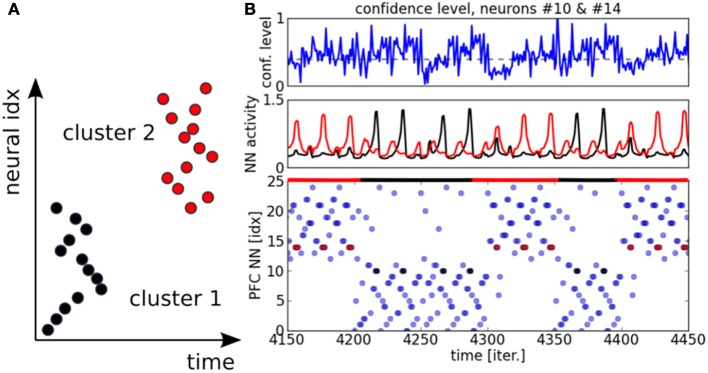
**Raster plot for PFC neurons**. In **(A)**, the PFC learns the particular temporal sequence from PPC outputs and it is sensitive to the temporal order of each unit in the sequence. In **(B)** on the top chart, the confidence level on the incoming signals from the PPC is plotted. The chart in the middle displays the neural activity for two neurons from the two distinct clusters. The neuron #10 in black (resp. cluster #1) and the neuron #14 in red (resp. cluster #2). The raster plot of the whole system is plotted in the chart below.

**Figure 10:**
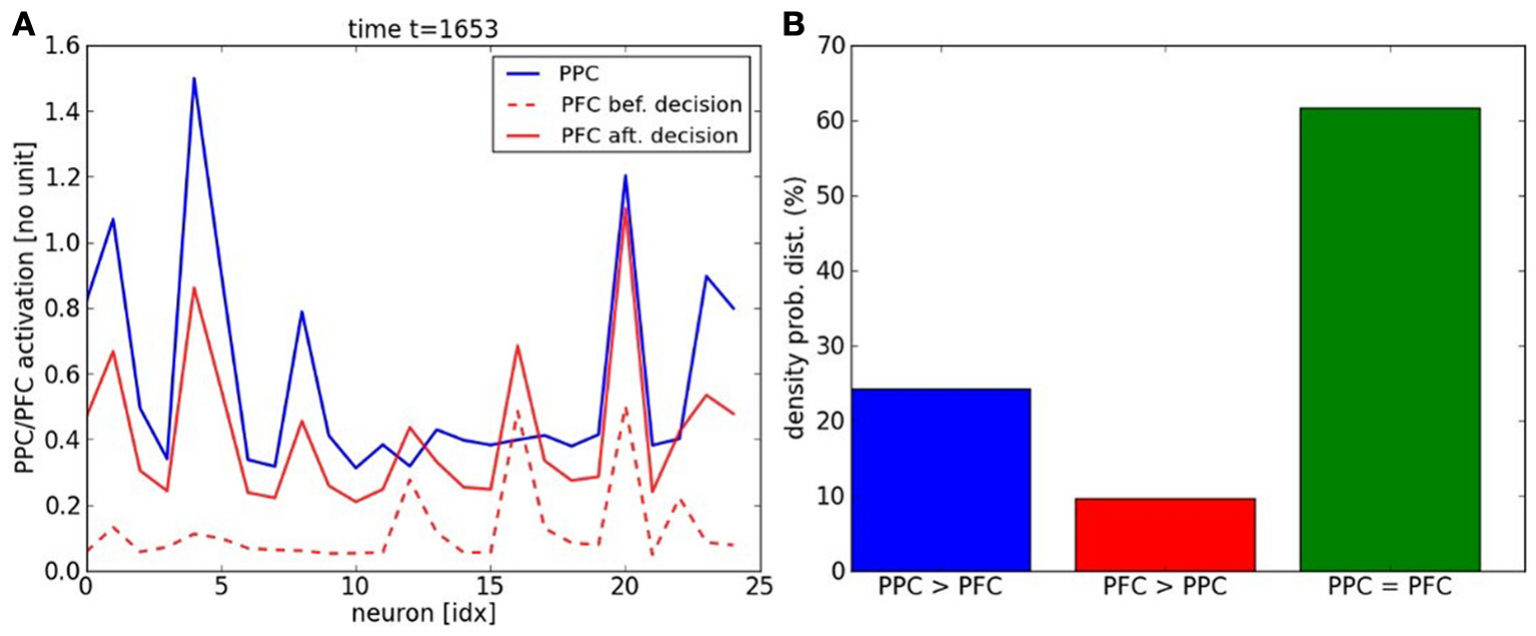
**PFC vs. PPC dynamics. (A)** The snapshot of the PPC/PFC dynamics at time *t* = 1653 show conflicting choices between the two maps, which correspond to a bifurcation point. After temporal integration, the PFC is processing the decision-making of a winner neuron different from the PPC choice. **(B)** Three PPC/PFC interactions occur, when PPC overwrites the values of PFC units, when PFC elicites its own values with respect to PPC and when both agree on the current predict. The PPC-PFC system works mostly in coherence from each other for 60% of the time (green bar) but in situations of conflict, the PPC overwrites twice the dynamics of the PFC network (blue bar) than the reverse (red bar).

The experiment is similar to the previous two-choices decision-making task, expect that the inputs follow now a temporal sequence within each map. When the inputs reach a particular point in the sequence–, a point to switch,– we proceed to a random choice between one or the two trajectories. As in the previous section, the learning phase for the PPC rapidly converges to the specialization of the two maps thanks to the ACC error-learning processing. Meanwhile, the PFC learns the temporal organization of the PPC outputs based on their sequential order, Figure 9A. We do not give to the PFC any information about length, the number of patterns or the order of the sequence. Besides, each firing neuron reinforces its links with the current pre-synaptic neurons; see the raster plot in Figure 9B. After the learning phase, each PFC neuron has learned to predict some portion of the sequence based on the past and current PFC activity. Their saliency to the current sequence is retranscribed in their amplitude level. We plot the activity level of the neurons #10 and #14 respectively in black and red in the second chart. This graph shows that their activity level gradually increases for period intervals of at least ten iterations till their firing. The points to switch are also learned by the network and they are observable when the variance of the neurons' activity level becomes low, which is also seen when the confidence level goes under 0.4; which corresponds to the dashed black line in the first chart. For instance, we plot the dynamics of the PPC neurons and of the PFC neurons during such situation in Figure 10A at time *t* = 1653. The neural dynamics of each map display different patterns and therefore, different decisions. The PPC activates more the neurons of the first map (the neurons with indices below thirteen in blue) whereas the PFC activates more the neurons of the second map (the neurons with indices above thirteen in dashed red). This shows that the PFC is not a purely passive system driven by the current activity in PPC/ACC. Besides, it learns also to predict the future events based on its past activity. The PFC fuses the two systems in its dynamics, and this is why it generates here a noisy output distribution due to the conflicting signals. We plot in Figure 10B the influence of PPC on the PFC dynamics. In 60% of the cases, the two systems agree to predict the current dynamics. This corresponds to the case of an automatic response when familiar dynamics are predicted. During conflicts, a prediction error is done by one of the two systems and in more cases the PPC dynamics, modulated by ACC, overwrite the values of the PFC units (blue bar). This situation occurs during a task switch for instance. At reverse, when PFC elicites its own values with respect to PPC (red bar), this situation occurs more when there is ambiguous sensory information that can be overpassed.

**Figure 11:**
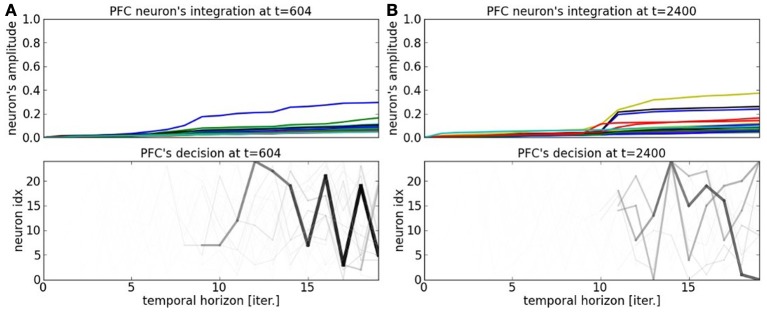
**PFC neuron's integration at time *t* = 604 and *t* = 2400. (A)** Depending on the current situation, a neuron will be more selective to one part of the sequence or to another. The earlier a sequence is detected, the farther the prediction of the trajectory. **(B)** At bifurcation points, the trajectories are fuzzier and several patterns are elicited.

In order to understand better the decision-making process within the PFC map, we display in Figures 11A,B the temporal integration done dynamically at each iteration within the network. Temporal integration means the process of summing the weights in Equation 2 at each iteration with respect to the current order. If the sequence order is well recognized, then the neuron's value goes high very rapidly, otherwise its value remains to a low value. As we explained it in the previous paragraph, each neuron is sensitive to certain patterns in the current sequence based on the synaptic links within the recurrent network. This is translated in the graph by the integration of bigger values. The spatio-temporal sequences they correspond to are darkened proportionally to their activation level. The higher is the activation level integration during the integration period, the faster is the anticipation of the sequence. We present the cases for a unambiguous pattern in Figure 11A and for an ambiguous sequence activity in Figure 11B. The case for a salient sequence recognition in Figure 11A indicates that the current part of the sequence is well estimated by at least one neuron, the winning neuron, which predicts well the sequence over twenty steps in advance, see the chart below. In comparison, the dynamics in Figure 11B show a more uniform probability distribution. This situation arises when a bifurcation point is near in the sequence, it indicates that the system cannot predict correctly the next steps of the sequence.

Considering the decision-making process per se, there is not a strict competition between the neurons, however, each neuron promotes one spatio-temporal sequence and one probability distribution. Therefore, we have within the system 25 spatio-temporal trajectories embedded. Based on the current situation, some neurons will detect better one portion of the sequence than others and the probability distribution will be updated in consequence to chain the actions sequentially, whereas other portions will collapse. The decision-making looks therefore similar to a self-organization process.

At this point, no inhibitory system has been implemented directly in PFC that would avoid a conflict in the sequence order. Instead, the PFC integrates the PPC signals with the ACC error signals. The temporal sequences done in the PPC to avoid the errors at the next moves are learned little by little by reinforcement in the PFC. These sequences become strategies for error avoidance and explorative search. Over time, they learn the prediction of reward and the prediction of errors (Schultz et al., 1997; Schultz and Dickinson, 2000).

**Figure 12:**
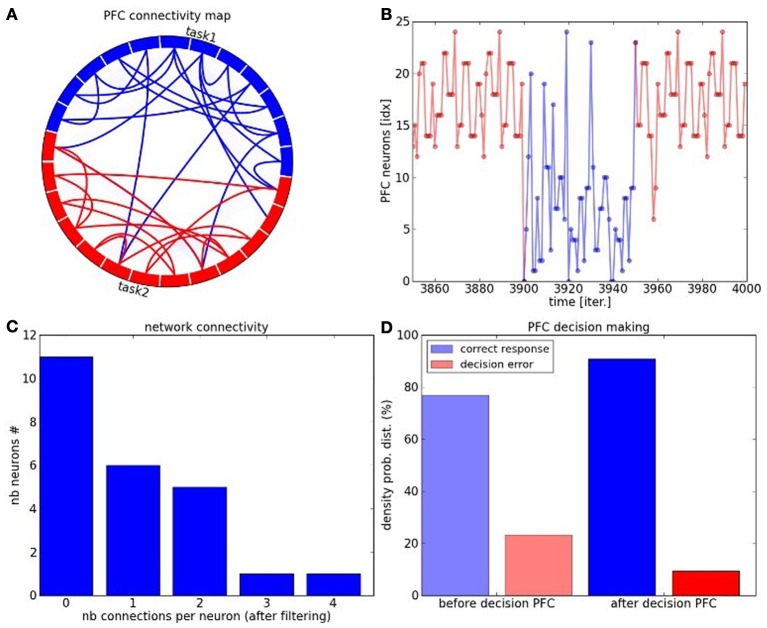
**PFC network analysis. (A)** Connectivity circle for the neurons of the PFC map. In blue are displayed the neurons belonging to cluster 1 and in red are displayed the neurons belonging to cluster 2. The number of links within each cluster (intra-map connectivity) is higher than the number of links between them (inter-map connectivity). Moreover, the number of highly connected neurons is also weak. these charateristic replicate the ones of complex systems and of small-world networks in particular. **(B)** Task switch is done through these hub-like neurons which can direct the trajectory from one or the other task. **(C)** The connectivity level per neurons within the network follows a logarithmic curve typical of complex networks, where the mostly connected neurons are also the fewer and the most critical with 4 distant connections. **(D)** The PFC network contributes to enhance the decision-making process in comparison to the PPC-ACC system due to the learning of the temporal sequence and to its better organization.

We perform some functional analysis on the PFC network in Figure 12. The connectivity circle in Figure 12A can permit to visualize the functional organization of the network at the neurons' level. We subdivide the PFC network into two sub-maps corresponding to the task dynamics in blue and red. We draw the strong intra-map connections between the neurons in the same color to their corresponding sub-maps as well as the strong inter-map connections between neurons of each map. Each neuron has a different connectivity in the network and the more it has connection the more it is central in the network. These neurons propagate information within and between the sub-maps, see Figure 12B. In complex systems terms, they are hub-like neurons from which different trajectories can be elicited. In decision-making, they are critical points for changing task. The density probability distribution plotted in Figure 12C shows that the maximum number of connections per neuron with strong synaptic weights reaches the number of four connections. Their number drastically diminishes with respect to the number of connections and their trend follows a logarithmic curve. These characteristics correspond the properties of small-world and scale-free networks.

In Figure 12D, we analyze the performance of the overall system when the PFC is added. The decision-making done in the PFC permits to decrease the error by a factor two: ten percents error in comparison to experiment 2. The prediction done in the recurrent map shows that the PFC is well organized to anticipate rewards and also task switch.

### 3.4. Experiment 4—robotic experiment on sensorimotor mapping and action selection

**Figure 13:**
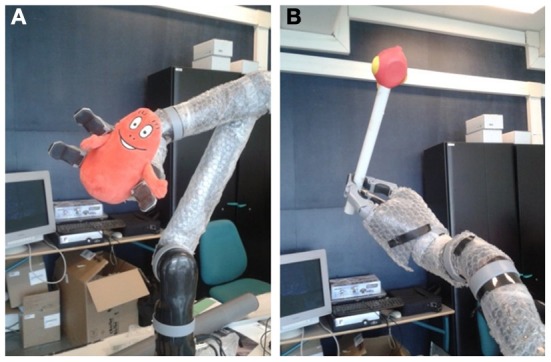
**Robot arm Kinova for task-set selection**. The two task-sets correspond to **(A)** the situation when it is moving its hand alone with the red target on its hand and **(B)** the situation when it is moving the stick on its hand with the red target on the tip of the tool.

We want to perform now a robotic experiment on action selection and decision making in the motor domain with a robotic arm of 6 degrees of freedom from the company Kinova; see Figure 13. We inspire ourself on the one hand from Wolpert's experiments on structural learning and representation of uncertainty in motor learning (Wolpert and Flanagan, 2010; Orban and Wolpert, 2011) and on the other hand from Iriki's experiments on the spatial adaptation following active tool-use (Iriki et al., 1996; Maravita and Iriki, 2004). Here, we attempt to learn different relations between states and motor commands when the robot controls its own arm alone and when it handles a tool. The question arises whether the robot will learn the structural affordances of the tool as a distinct representation or, instead, as part of its limb's representation (Cothros et al., 2006; Kluzik et al., 2008). Iriki et al. (1996) reported that bimodal-cell visual receptive fields (vRFs) show spatial adaptation following active tool-use, but not passive holding. The spatial estimation of its own body limits—that is, its body image,—is different depending on the attention to the tool. The goal is therefore to estimate properly the current situation on which the robot is, which means handling a stick or not, actively or passively.

In our framework, we expect that the errors of spatial estimation on the end-point can be gradually learned and that sensorimotor mapping will change with respect to the tasks the robot has to perform (Wolpert and Flanagan, 2010; Orban and Wolpert, 2011). Figures 13A,B display the arm robot when it holds a salient toy and when it handles a stick with the toy at its end-point. In this experiment, a fixed camera is mapping the x-y coordinates of the salient points (i.e., the toy) while the robot moves its arm around its elbow; we make the note that we circumscribe the problem to two modalities only in order to control just one articulation with respect to the Y axis in the camera.

In the previous experiments, we did not exploit specifically the properties of the gain-field neurons for mapping sensorimotor transformation. Here instead, we use the gain-field mechanism to combine the visuomotor information into the PPC system for the two contexts. With respect to the task, the PPC output maps will learn the specific amplitude of the gain-field neurons corresponding to the specific visuomotor relationships (Holmes et al., 2007).

**Figure 14:**
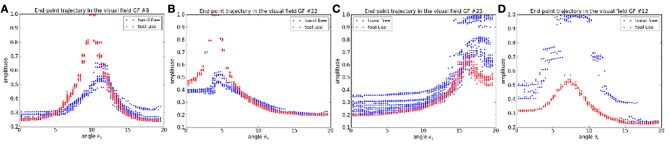
**Dynamics of the gain-field neurons relative to the task. (A–D)** In blue, the robot moves its hand freely. In red, the robot is handling the tool. Depending on what the GF neurons have learned, their peak level will diminish or increase when changing the task (i.e., using a tool).

For instance, we plot in Figures 14A–D the activity level of four different gain-field neurons relative to the motor angle θ_0_ of the robot arm. The blue dots represent the situation when it weaves the hand in front of the camera and the red dots represent the situation when it is handling the tool. As the gain-field neurons learn the specific relationship between certain values of the XY coordinates of the end-point effector and the motor angle θ_0_, this value is modulated when the robot arm uses the stick; see resp. Figures 14A–D. The visuo-motor translation in the XY plane when the robot is handling the tool produces a gain modulation that decreases or increases the neurons' activity level.

**Figure 15:**
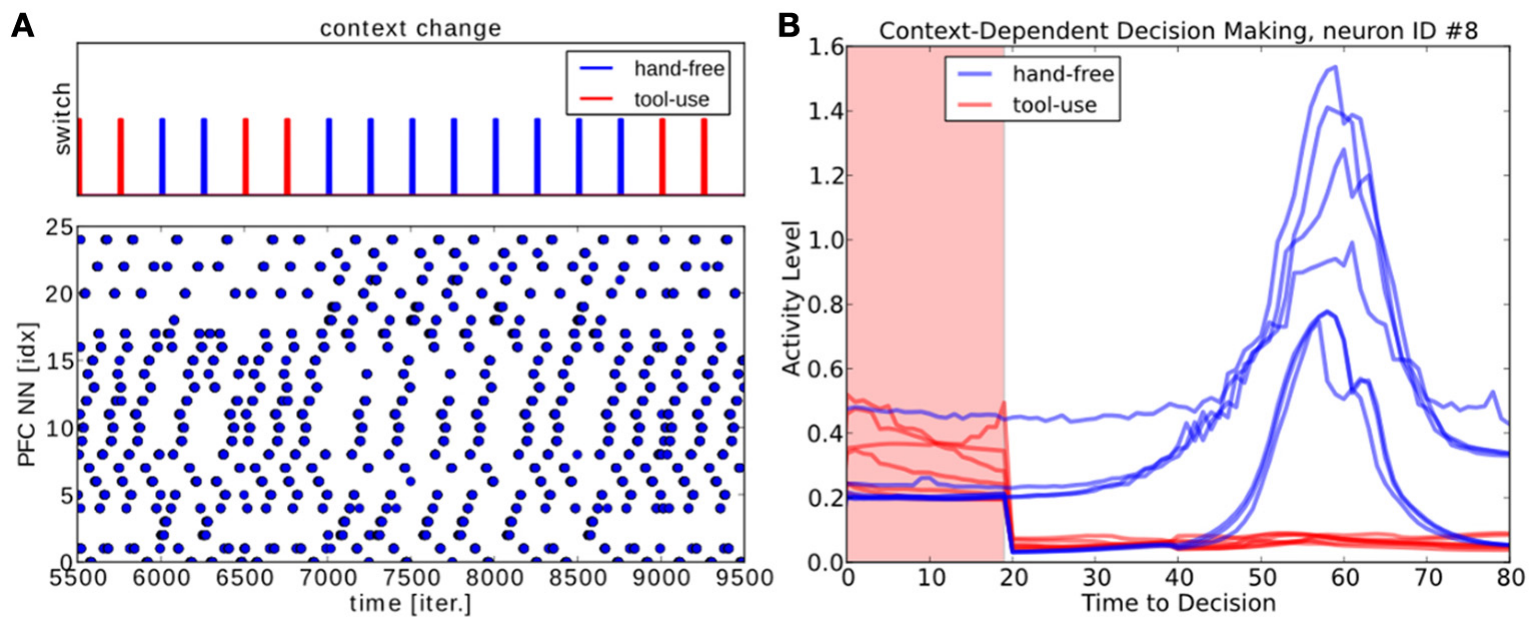
**PFC Attention decision during contextual change, hand-free or tool-use. (A)** we expose to the PFC dynamics some incomplete patterns for a short period of time of 20 iterations, every 500 iterations. The PFC is capable to switch to the reconstruct back the missing part of the spatio-temporal sequence; in blue for hand-free and in red for tol-use. **(B)** Neural activity for one neuron when one of the two contexts is set.

Hence, the visuomotor coordination changes instantaneously the GF neurons' activity level relative to the current task set and the PPC is dynamically driven by the input activity (not displayed). The neural activity in the PFC map, instead, can evolve autonomously and independently with respect to the input activity, even if the PPC dynamics are presented for a short exposure; this behavior is displayed in the raster plot in Figure 15A. When we expose the PFC neurons to the PPC dynamics for a small period of time—20 iterations every 500 iterations (the segments on the top chart),—the network is able to reconstruct dynamically the rest of the ongoing sequence; see Figure 15B. For instance, the neuron #8 is selective to the particular context of hand-free (blue lines). The contextual information is maintainted as a stable pattern of the neural activity in the working memory and the contexts are accessible and available for influencing the ongoing processing. As a recurrent network, the PFC behaves similarly to a working memory. It embeds the two different strategies depending on the context, even in presence of incomplete inputs and can select to attend or not to the tool.

## 4. Discussion

The ability to learn the structure of actions and to select on the fly the proper one given the current task is one great leap in infants cognition. During development, infants learn to differentiate their motor behaviors relative to various contexts by exploring and identifying the correct structures of causes and effects that they can perform by trial and errors. This behavior corresponds to an intrinsic motivation, a mechanism that is argued to drive cognitive development. Besides, Karen Adolph emphasizes the idea of “learning-to-learn” in motor development, an expression akin to Harlow that appears in line with the one of intrinsic motivation. She proposes that two learning mechanisms embody this concept during the development of the motor system—, respectively an associative memory and a category-based memory,– and that the combination of these two learning systems is involved in this capacity of learning-to-learn. Braun et al. (2010) foster a similar concept and suggest that motor categorization requires 1) a critic for learning the structure, i.e., an error-based system, and 2) a learning system that will learn the conditional relationships between the incoming variables; which means, the parameters of the task. They argue that once these parameters are found, it is easier to transfer knowledge from one initial task to many others. All-in-all, we believe that these different concepts on structural learning are important to scaffold motor development and to have intrinsic motivation in one system. Thus the question arises what are the neural mechanisms involved in structural learning and in flexible behaviors?

To investigate this question, we have modeled an architecture that attempts to replicate the functional organization of the fronto-parietal structures, namely, a sensorimotor mapping system, an error-processing system and a reward predictor (Platt and Glimcher, 1999; Westendorff et al., 2010). The fronto-parietal cortices are involved in activities related to observations of alternatives and to action planning, and the anterior cyngulate cortex is a part of this decision-making network. Each of these neural systems contribute to one functional part of it. The ACC system is processing the error-negativity reward to the PPC maps for specialization and to the PFC network for reward prediction. The PPC network organizes the sensorimotor mapping for different tasks whereas the PFC learns the spatio-temporal patterns during the act.

In particular, the PPC is organized around the mechanism of gain-modulation where the gain-fields neurons combine the sensory inputs from each other. We suggest that the mechanism of gain-modulation can implement the idea of structural learning in motor tasks proposed by Braun and Wolpert (Braun et al., 2009, 2010). In their framework, the gain-field neurons can be seen as basis functions and as the parameters of the learning problem. It is interesting to note that Braun and al. make a parallel with the bayesian framework, which has been also proposed to describe the gain-field mechanism. For instance, Deneve explains the computational capabilities of gain-fields in the context of the bayesian framework to efficiently represent the joint distribution of a set of random variables (Denève and Pouget, 2004).

Parallely, we used three specific intrinsic mechanisms for enhancing structural learning: the rank-order coding algorithm, the cortical plasticity and an error-based reward. For instance, the rank-order coding algorithm was used to emulate efficiently the so-called spike timing-dependent plasticity to learn spatio-temporal sequences in a recurrent network (Bi and Poo, 1998; Abbott and Nelson, 2000). The PFC system exploits their properties for self-organizing itself by learning the sequences of each task as well as the switch points. PFC neurons learn specific trajectories and at each iteration, a competition process is at work to promote the new steps of the ongoing sequence. Besides, cortical plasticity was modeled in PPC maps with an activity-dependent learning mechanism that promotes the rapid learning of novel (experienced-based) tasks and the stabilization of the old ones. An advantageous side-effect of this mechanism is that PPC neurons become context-dependent, which is a behavior observed also in the reaching neurons of the parieto-motor system, the so-called mirror neurons (Gallese et al., 1996; Brozovic et al., 2007). The results found on cortical plasticity are in line with observations on the rapid adaptation of the body image and of the motor control. Wolpert observed that the motor system incorporates a slow learning mechanism along a fast one for the rapid formation of task sets (Wolpert and Flanagan, 2010). The cortical plasticity is also influenced by an error-based system in ACC that reshape the PPC dynamics with respect to the task. The negative reward permits to inhibit the wrong dynamics but not to elicite the correct ones. Those ones are gradually found by trial and errors, which replicate an exploration process.

We believe that these different mechanisms are important for incremental learning and intrinsic motivation. However, many gaps remain. For instance, a truly adaptive system should show more flexibility during familiar situations than during unfamiliar ones. Retranscribed from Adolph and Joh (2005), a key to flexibility is (1) to refrain from forming automatic responses and (2) to identify the critical features that allow online problem solving to occur. This ability is still missing in current robots. In the context of problem solving in tool-use, Fagard and O'Regan emphasizes the similar difficulty for infants to use a stick for reaching a toy. They also observe that below a certain age, attention is limited to one object only as they just cannot “hold in mind” the main goal in order to perform one subgoal (Fagard et al., 2012; Rat-Fischer et al., 2012). Above this period, however, Fagard and O'Regan observe an abrupt transition in their behaviors when they became capable to relate two actions at a time, to plan consecutive actions and to use recursion. They hypothesize that after 16 months, infants are able to enlarge their focus of attention to two objects simultaneously and to “bufferize” the main goal. We make a parallel with the works of Koechlin and colleagues Koechlin et al. (2003); Collins and Koechlin (2012) who attribute a monitoring role to the frontal cortex for maintaining the working memory relative to the current tasks and for prospecting the different action sequences or episodic memories (Koechlin and Summerfield, 2007), which will be our next steps.

### Conflict of interest statement

The authors declare that the research was conducted in the absence of any commercial or financial relationships that could be construed as a potential conflict of interest.

## References

[B1] Abbott L. Nelson S. (2000). Synaptic plasticity: taming the beast. Nat. Neurosci. 3, 1178–1182 10.1038/81453 11127835

[B2] Adolph K. (2008). Learning to move. Curr. Dir. Psychol. Sci. 17, 213–218 10.1111/j.1467-8721.2008.00577.x 19305638 PMC2658759

[B3] Adolph K. Joh A. (2005). Multiple learning mechanisms in the development of action, in Paper presented to the Conference on Motor Development and Learning. Vol. 33, eds Lockman J. Reiser J. Nelson C. A. (Murcia).

[B4] Adolph K. Joh A. (2009). Multiple Learning Mechanisms in the Development of Action. New York, NY: Oxford University Press

[B5] Andersen R. (1997). Multimodal integration for the representation of space in the posterior parietal cortex. Philos. Trans. R. Soc. Lond. B Biol. Sci. 353, 1421–1428 10.1098/rstb.1997.0128 9368930 PMC1692052

[B6] Andersen R. Cui H. (2009). Intention, action planning, and decision making in parietal-frontal circuits. Neuron 63, 568–583 10.1016/j.neuron.2009.08.028 19755101

[B7] Barto A. (1995). Adaptive critics and the basal ganglia, in Models of Information Processing in the Basal Ganglia eds Houk J. Davis J. Beiser D. (Cambridge, MA: MIT Press), 215–232

[B8] Baumann M. Fluet M. C. Scherberger H. (2009). Context-specific grasp movement representation in the macaque anterior intraparietal area. J. Neurosci. 29, 6436–6448 10.1523/JNEUROSCI.5479-08.2009 19458215 PMC6665886

[B9] Bi G. Poo M. (1998). Activity-induced synaptic modifications in hippocampal culture, dependence of spike timing, synaptic strength and cell type. J. Neursci. 18, 10464–10472 9852584 10.1523/JNEUROSCI.18-24-10464.1998 PMC6793365

[B10] Botvinick M. Braver T. Barch D. Carter C. Cohen J. (2001). Conflict monitoring and cognitive control. Psychol. Rev. 108, 624–652 10.1037/0033-295X.108.3.624 11488380

[B11] Braun D. Aertsen A. Wolpert D. Mehring C. (2009). Motor task variation induces structural learning. Curr. Biol. 19, 352–357 10.1016/j.cub.2009.01.036 19217296 PMC2669412

[B12] Braun D. Mehring C. Wolpert D. (2010). Structure learning in action. Behav. Brain Res. 206, 157–165 10.1016/j.bbr.2009.08.031 19720086 PMC2778795

[B13] Brozovic M. Gail A. Andersen R. (2007). Gain mechanisms for contextually guided visuomotor transformations. J. Neurosci. 27, 10588–10596 10.1523/JNEUROSCI.2685-07.2007 17898230 PMC6673148

[B14] Churchland A. Ditterich J. (2012). New advances in understanding decisions among multiple alternatives. Curr. Opin. Neurobiol. 22, 920–926 10.1016/j.conb.2012.04.009 22554881 PMC3422607

[B15] Collins A. Koechlin E. (2012). Reasoning, learning, and creativity: frontal lobe function and human decision-making. PLoS Biol. 10:e1001293 10.1371/journal.pbio.1001293 22479152 PMC3313946

[B16] Cothros N. Wong J. Gribble P. (2006). Are there distinct neural representations of object and limb dynamics? Exp. Brain Res. 173, 689–697 10.1007/s00221-006-0411-0 16525798

[B17] Denève S. Pouget A. (2004). Bayesian multisensory integration and cross-modal spatial links. J. Neurophysiol. 98, 249–258 10.1016/j.jphysparis.2004.03.011 15477036

[B18] Diamond A. (1985). Development of the ability to use recall to guide action, as indicated by infants' performance on a-not-b. Child Dev. 74, 24–40 4042750

[B19] Diamond A. (1990). Rate of maturation of the hippocampus and the developmental progression of children's performance on the delayed non-matching to sample and visual paired comparison tasks. Ann. N.Y. Acad. Sci. 608, 394–426; discussion: 426–433. 10.1111/j.1749-6632.1990.tb48904.x 2127514

[B20] Fagard J. Rat-Fischer L. O'Regan J. (2012). Comment le bb accde-t-il la notion d'outil? Enfance 64, 73–84 10.4074/S0013754512001085

[B21] Fluet M. Baumann M. Scherberger H. (2010). Context-specific grasp movement representation in macaque ventral premotor cortex. J. Neurosci. 30, 15175–1518 10.1523/JNEUROSCI.3343-10.2010 21068323 PMC6633833

[B22] Fuster J. (2001). The prefrontal cortexan update: Time is of the essence. Neuron 30, 319–333 10.1016/S0896-6273(01)00285-9 11394996

[B23] Gallese V. Fadiga L. Fogassi L. Rizzolatti G. (1996). Action recognition in the premotor cortex. Brain 119, 593–609 10.1093/brain/119.2.593 8800951

[B24] Guerin F. Kruger N. Kraft D. (2013). A survey of the ontogeny of tool use: from sensorimotor experience to planning. Aut. Ment. Dev. IEEE Trans. 5, 18–45 10.1109/TAMD.2012.2209879

[B25] Harlow H. (1949). The formation of learning sets. Psychol. Rev. 56, 51–65 10.1037/h0062474 18124807

[B26] Holmes N. Sanabria D. Calvert G. Spence C. (2007). Tool-use: capturing multisensory spatial attention or extending multisensory peripersonal space? Cortex 43, 469–489 10.1016/S0010-9452(08)70471-4 17533769 PMC1885399

[B27] Holroyd C. Coles M. (2002). The neural basis of human error processing: reinforcement learning, dopamine, and the error-related negativity. Psychol. Rev. 109, 679–709 10.1037/0033-295X.109.4.679 12374324

[B28] Holtmaat A. Svoboda K. (2009). Experience-dependent structural synaptic plasticity in the mammalian brain. Nat. Rev. Neurosci. 10, 647–665 10.1038/nrn2699 19693029

[B29] Iriki A. Tanaka M. Iwamura Y. (1996). Coding of modified body schema during tool use by macaque postcentral neurones. Neuroreport 7, 2325–2330 10.1097/00001756-199610020-00010 8951846

[B30] Izhikevich E. Gally A. Edelman G. (2004). Spike-timing dynamics of neuronal groups. Cereb. Cortex 14, 933–944 10.1093/cercor/bhh053 15142958

[B31] Johnson M. (2012). Executive function and developmental disorders: the flip side of the coin. Trends Cogn. Sci. 16, 454–457 10.1016/j.tics.2012.07.001 22835639

[B32] Kaplan F. Oudeyer P.-Y. (2007). In search of the neural circuits of intrinsic motivation. Front. Neurosci. 1, 225–236 10.3389/neuro.01.1.1.017.2007 18982131 PMC2518057

[B33] Khamassi M. LallÃl'e S. Enel P. Procyk E. Dominey P. (2011). Robot cognitive control with a neurophysiologically inspired reinforcement learning model. Front. Neurorobot. 5, 1–14 10.3389/fnbot.2011.00001 21808619 PMC3136731

[B34] Kluzik J. Diedrichsen J. Shadmehr R. Bastian A. (2008). Reach adaptation: what determines whether we learn an internal model of the tool or adapt the model of our arm? J. Neurophysiol. 100, 1455–1464 10.1152/jn.90334.2008 18596187 PMC2544452

[B35] Koechlin E. Ody C. Kouneilher F. (2003). The architecture of cognitive control in the human prefrontal cortex. Science 302, 1181–1185 10.1126/science.1088545 14615530

[B36] Koechlin E. Summerfield C. (2007). An information theoretical approach to prefrontal executive function. Trends Cogn. Sci. 11, 229–235 10.1016/j.tics.2007.04.005 17475536

[B37] Kohonen T. (1982). Self-organized formation of topologically correct feature maps. Biol. Cybern. 43, 59–69 10.1007/BF00337288

[B38] Lockman J. (2000). A perception-action perspective on tool use development. Child Dev. 71, 137–144 10.1111/1467-8624.00127 10836567

[B39] Maravita A. Iriki A. (2004). Tools for the body (schema). Trends Cogn. Sci. 8, 79–86 10.1016/j.tics.2003.12.008 15588812

[B40] Orban G. Wolpert D. (2011). Representations of uncertainty in sensorimotor control. Curr. Opin. Neurobiol. 21, 1–7 10.1016/j.conb.2011.05.026 21689923

[B41] Pitti A. Blanchard A. Cardinaux M. Gaussier P. (2012). Gain-field modulation mechanism in multimodal networks for spatial perception, 12th IEEE-RAS International Conference on Humanoid Robots Nov.29-Dec.1, 2012. Business Innovation Center Osaka, 297–302

[B42] Pitti A. Kuniyoshi Y. (2011). Modeling the cholinergic innervation in the infant cortico-hippocampal system and its contribution to early memory development and attention, Proceedings of the International Joint Conference on Neural Networks (IJCNN11), (San Jose, CA), 1409–1416 10.1109/IJCNN.2011.6033389

[B43] Platt M. Glimcher P. (1999). Neural correlates of decision variables in parietal cortex. Nature 400, 233–238 10.1038/22268 10421364

[B44] Pouget A. Snyder L. (2000). Computational approaches to sensorimotor transformations. Nat. Neurosci. 3, 1192–1198 10.1038/81469 11127837

[B45] Rat-Fischer L. O'Regan J. Fagard J. (2012). The emergence of tool use during the second year of life. J. Exp. Child Psychol. 113, 440–446 10.1016/j.jecp.2012.06.001 22789968

[B46] Rougier N. Boniface Y. (2011). Dynamic self-organising map. Neurocomputing 74, 1840–1847 10.1016/j.neucom.2010.06.034

[B47] Salinas E. Thier P. (2000). Gain modulation: a major computational principle of the central nervous system. Neuron 27, 15–21 10.1016/S0896-6273(00)00004-0 10939327

[B48] Schöner G. Dineva E. (2007). Dynamic instabilities as mechanisms for emergence. Dev. Sci. 10, 69–74 10.1111/j.1467-7687.2007.00566.x 17181702

[B49] Schultz W. Dayan P. Montague P. (1997). A neural substrate of prediction and reward. Annu. Rev. Neurosci. 275, 1593–1599 9054347 10.1126/science.275.5306.1593

[B50] Schultz W. Dickinson A. (2000). Neuronal coding of prediction errors. Annu. Rev. Neurosci. 23, 473–500 10.1146/annurev.neuro.23.1.473 10845072

[B51] Singh S. Lewis R. Barto A. Sorg J. (2010). Intrinsically motivated reinforcement learning: an evolutionary perspective. IEEE Trans. Autonom. Mental Dev. 2, 70–82 10.1109/TAMD.2010.2051031

[B52] Smith L. Thelen E. Titzer R. McLin D. (1999). Knowing in the context of acting: The task dynamics of the a-not-b error. Psychol. Rev. 106, 235–260 10.1037/0033-295X.106.2.235 10378013

[B53] Stricanne B. Andersen R. Mazzon P. (1996). Eye-centered, head-centered, and intermediate coding of remembered sound locations in area lip. J. Neurophysiol. 76, 2071–2076 8890315 10.1152/jn.1996.76.3.2071

[B54] Tenenbaum J. Kemp C. Griffiths T. Goodman N. (2011). How to grow a mind: statistics, structure, and abstraction. Science 331, 1279–1285 10.1126/science.1192788 21393536

[B55] Thorpe S. Delorme A. Van Rullen R. (2001). Spike-based strategies for rapid processing. Neural Netw. 14, 715–725 10.1016/S0893-6080(01)00083-1 11665765

[B56] Vaesen K. (2012). The cognitive bases of human tool use. Behav. Brain Sci. 35, 203–262 10.1017/S0140525X11001452 22697258

[B57] Van Rullen R. Gautrais J. Delorme A. Thorpe S. (1998). Face processing using one spike per neurone. Biosystems 48, 229–239 10.1016/S0303-2647(98)00070-7 9886652

[B58] Van Rullen R. Thorpe S. (2002). Surfing a spike wave down the ventral stream. Vision Res. 42, 2593–2615 10.1016/S0042-6989(02)00298-5 12446033

[B59] Warren J. Harlow H. (1952). Learned discrimination performance by monkeys after prolonged postoperative recovery from large cortical lesions. J. Comp. Physiol. Psychol. 45, 119–126 10.1037/h0055350 14927754

[B60] Westendorff S. Klaes C. Gail A. (2010). The cortical timeline for deciding on reach motor goals. J. Neurosci. 30, 5426–5436 10.1523/JNEUROSCI.4628-09.2010 20392964 PMC6632761

[B61] White O. Diedrichsen J. (2013). Flexible switching of feedback control mechanisms allows for learning of different task dynamics. PLoS ONE 8:e54771 10.1371/journal.pone.0054771 23405093 PMC3566087

[B62] Wolpert D. Diedrichsen J. Flanagan J. (2011). Principles of sensorimotor learning. Nat. Rev. Neurosci. 12, 739–751 10.1038/nrn3112 22033537

[B63] Wolpert D. Flanagan J. (2010). Motor learning. Curr. Biol. 20, R467–R472 10.1016/j.cub.2010.04.035 20541489

[B64] Xu T. Yu X. Perlik A. Tobin W. Zweig J. Tennant K. (2009). Rapid formation and selective stabilization of synapses for enduring motor memories. Nature 462, 915–919 10.1038/nature08389 19946267 PMC2844762

[B65] Yokoyama C. Tsukada H. Watanabe Y. Onoe H. (2005). A dynamic shift of neural network activity before and after learning-set formation. Cereb. Cortex 15, 796–801 10.1093/cercor/bhh180 15371296

[B66] Ziv N. Ahissar E. (2009). New tricks and old spines. Nature 462, 859–861 10.1038/462859a 20016588

